# Comparison of mechanisms and transferability of outcomes of SGLT2 inhibition between type 1 and type 2 diabetes

**DOI:** 10.1002/edm2.129

**Published:** 2020-04-24

**Authors:** Oliver Schnell, Paul Valensi, Eberhard Standl, Antonio Ceriello

**Affiliations:** ^1^ Forschergruppe Diabetes e. V. Munich Germany; ^2^ Department of Endocrinology Diabetology Nutrition APHP, Jean VERDIER Hospital, Paris Nord University, CINFO, CRNH‐IdF Bondy France; ^3^ Department of Cardiovascular and Metabolic Diseases IRCCS MultiMedica Sesto San Giovanni (MI) Italy

**Keywords:** Diabetes, mechanisms of SGLT‐2 inhibition, SGLT‐2 inhibitor, transferability, type 2 diabetes; type 1 diabetes

## Abstract

Diabetes mellitus (DM) is a major chronic disease with ever‐increasing prevalence and a variety of serious complications for persons with DM, such as cardiovascular and/or renal complications. New glucose‐lowering therapies like DPP‐4 inhibitors, GLP‐1 receptor agonists, and SGLT‐2 inhibitors have undergone cardiovascular outcome trials (CVOTs) for type 2 diabetes (T2DM), as by the guidance of the FDA. However, CVOTs for type 1 diabetes (T1DM) are generally lacking. Both, persons with T1DM and T2DM, are burdened with a high incidence of cardiovascular and renal disease such as atherosclerotic cardiovascular disease (ASCVD) and diabetic kidney disease (DKD). Although pathologies of the two types of diabetes cannot be compared, similar mechanisms and risk factors like sex, hyperglycaemia, hypertension, endothelial damage and (background) inflammation have been identified in the development of CVD and DKD in T1DM and T2DM. Recent CVOTs in T2DM demonstrated that SGLT‐2 inhibitors, besides exerting a glucose‐lowering effect, have beneficial effects on cardiovascular and renal mechanisms. These mechanisms are reviewed in detail in this manuscript and evaluated for possible transferability to, and thus efficacy in, T1DM. Our review of current literature suggests that SGLT‐2 inhibitors have cardioprotective benefits beyond their glucose‐lowering effects. As this mainly has been observed in CVOTs in T2DM, further investigation in the adjunctive therapy for type 1 diabetes is suggested.

## Graphical abstract

Both, persons with T1DM and T2DM, are burdened with a high incidence of cardiovascular and renal disease such as atherosclerotic cardiovascular disease (ASCVD) and diabetic kidney disease (DKD). Although pathologies of the two types of diabetes cannot be compared, similar mechanisms and risk factors like sex, hyperglycaemia, hypertension, endothelial damage and inflammation have been identified in the development of ASCVD and DKD in T1DM and T2DM. Recent CVOTs in T2DM demonstrated that SGLT‐2 inhibitors, besides exerting a glucose‐lowering effect, have beneficial effects on cardiovascular and renal mechanisms. These mechanisms are reviewed in detail in this manuscript and evaluated for possible transferability to, and thus efficacy in, T1DM.

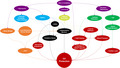

## 1 INTRODUCTION

According to the 2019 Atlas of the International Diabetes Federation, an estimated 463.0 million adults currently live with diabetes mellitus (DM), accounting for both, persons with type 1 (T1DM) and type 2 diabetes (T2DM) between the ages of 20‐79.^1^ Numbers are predicted to rise to approximately 700.2 million patients with DM by 2045.^1^ In 2019, approximately 1.11 million children and adolescent patients between the age of 0‐19 years were estimated to be burdened with T1DM worldwide.^1^

While mechanisms of pathogenesis differ between T1DM and T2DM, both diseases confer substantial risk of comorbidities such as diabetic kidney disease (DKD), retinopathy, neuropathy, cardiovascular disease (CVD), and cerebrovascular disease. T1DM usually develops early in patients’ lives (childhood or adolescence) and is classified as autoimmune disease with yet uncertain origin, resulting in systemic insulin deprivation, hyperglycaemia and a bias for the development of ketoacidosis. In T2DM, the body's cells develop an insulin resistance (IR) and beta‐cell dysfunction over time and systemic hyperglycaemia occurs. A variety of treatment options aside from insulin have been developed especially for T2DM, including sodium/glucose co‐transporter 2 inhibitors (SGLT‐2is). SGLT‐2is aim at reducing hyperglycaemia by removal of excess glucose through the kidneys by increasing glycosuria. Different SGLT‐2is have undergone extensive evaluation in various cardiovascular outcome trials (CVOTs); however, as these studies only address patients with T2DM, the late‐breaking question has been if SGLT‐2is will confer equal or varying benefits when used as an adjunct treatment to insulin in T1DM. While first efficacy and safety studies in T1DM^2^, ^3^, ^4^, ^5^, ^6^, ^7^, ^8^ have shown promising results, large‐scale and long‐term studies to assess the actual extent of cardiovascular (CV) and renal benefits in T1DM are currently lacking.

This review aims at highlighting and comparing mechanisms in the genesis of the two prominent diabetic comorbidities CVD and DKD between T1DM and T2DM. Common features of CV and renal disease in T1DM and T2DM include risk factors like hyperglycaemia, blood pressure (BP), body weight and associated effects. In addition, other mechanisms affected by SGLT‐2is like the renal tubuloglomerular feedback and its implications, cardiac substrate utilization in diabetes, the impact of inflammation, and direct effects on cardiac function are discussed. The review aims at comparing the learnings from SGLT‐2i CVOTs in T2DM to T1DM on a mechanistic basis and, to best current knowledge, elaborate why and when it could be particularly advantageous to consider SGLT‐2i use as an adjunct therapy to insulin in patients with T1DM, especially with respect to concomitant CVD and renal outcomes.

## 2 SGLT‐2 INHIBITION: THERAPEUTIC PRINCIPLE

Sodium/glucose co‐transporters (SGLTs) are located at the apical membrane of the renal tubules and reabsorb filtered glucose by actively transporting glucose together with sodium against a concentration gradient, while the basolaterally located glucose transporters (GLUTs) mediate the transport of glucose back into the circulation along an existing concentration gradient.^9^, ^10^ The SGLT‐family is represented by two members in the kidneys, SGLT‐1 (late S2/S3 segment of the renal tubule) and SGLT‐2 (S1 segment of the renal tubule). Even though, next to the liver, the kidneys have substantial gluconeogenic activity, reabsorption of glucose is the kidneys’ main contribution to systemic glucose homoeostasis as they metabolize large parts of their glucose output themselves.^11^ The vast majority of filtered glucose (more than 99%) is reabsorbed in the renal tubular system: up to 97% of filtered glucose is reabsorbed in the proximal renal tubule by SGLT‐2, the remaining ≈3% of filtered glucose is reabsorbed by SGLT‐1.^12^, ^13^ Of note, when blood glucose levels are increased, the amount of glucose filtered by the kidneys is enlarged.^12^ Normally filtering around 180g of glucose per day, with a maximum reabsorptive capacity of around 430‐500g/24h, it has been shown in patients with DM that the maximal reabsorptive capacity of the kidneys was increased by up to 20% to approximately 500‐600 g/24 h.^12^

Inhibition of SGLT‐2 efficiently decreases the amount of glucose and sodium reabsorbed and fed back into systemic circulation. However, with SGLT‐2 inhibition alone, it has been shown that only around 50%‐60% of filtered glucose are excreted in normoglycaemic individuals, as significant downstream compensation is achieved by an increase in the expression or activity of renal SGLT‐1 in response to blockage of SGLT‐2.^13^, ^14^ Factors which influence the efficacy of SGLT‐2i–mediated blood glucose‐lowering are (1) renal function (decreasing glucose‐lowering effect with decreasing renal function)^15^; (2) the degree of hyperglycaemia which leads to a.) upregulation of renal SGLT‐2 expression and b.) an increase in glucose reabsorption, hence enhancement of glycosuria by SGLT‐2 inhibition; (3) an increase in gluconeogenesis (likely hepatic^12^) upon SGLT‐2 inhibition, increasing overall blood glucose levels ^16^, ^17^; and (4.) perhaps variances in the regulation of the expression of renal SGLT‐1 in response to DM, or a luminal translocation of GLUT2.^12^ Of eminent and central importance for the concept of clinical use of SGLT‐2is as an adjunct therapy of T1DM is the converse argument of increased SGLT‐2i efficacy with increased blood glucose: while SGLT‐2i efficacy increases with augmented blood glucose (due to increased amounts of glucose reabsorbed in the kidneys), conversely, when blood glucose is decreased, the increase in urinary glucose excretion is attenuated, effectively reducing the risk of hypoglycaemia.^18^

The major risk associated with administration of SGLT‐2is, particularly in T1DM, has been shown to be augmented rates of diabetic ketoacidosis (DKA)—also in euglycaemic range.^19^ This is owed to the fact that SGLT‐2i‐induced glycosuria substantially lowers blood glucose—resulting in reduced availability of carbohydrate substrate, often in conjunction with a reduction in insulin dose as demonstrated in several trials in T1DM^2^, ^3^, ^4^, ^6^, ^7^, ^8^—thus predisposes to increased ketogenesis and serum ketone levels.^19^ All long‐term trial programmes with SGLT‐2is as an adjunct therapy to insulin in T1DM showed an increase in rates of DKA.^2^, ^3^, ^4^, ^6^, ^7^ These facts highlight the eminent need for proper patient selection and patient education by specialists in order to balance risks and benefits.

**Figure 1:**
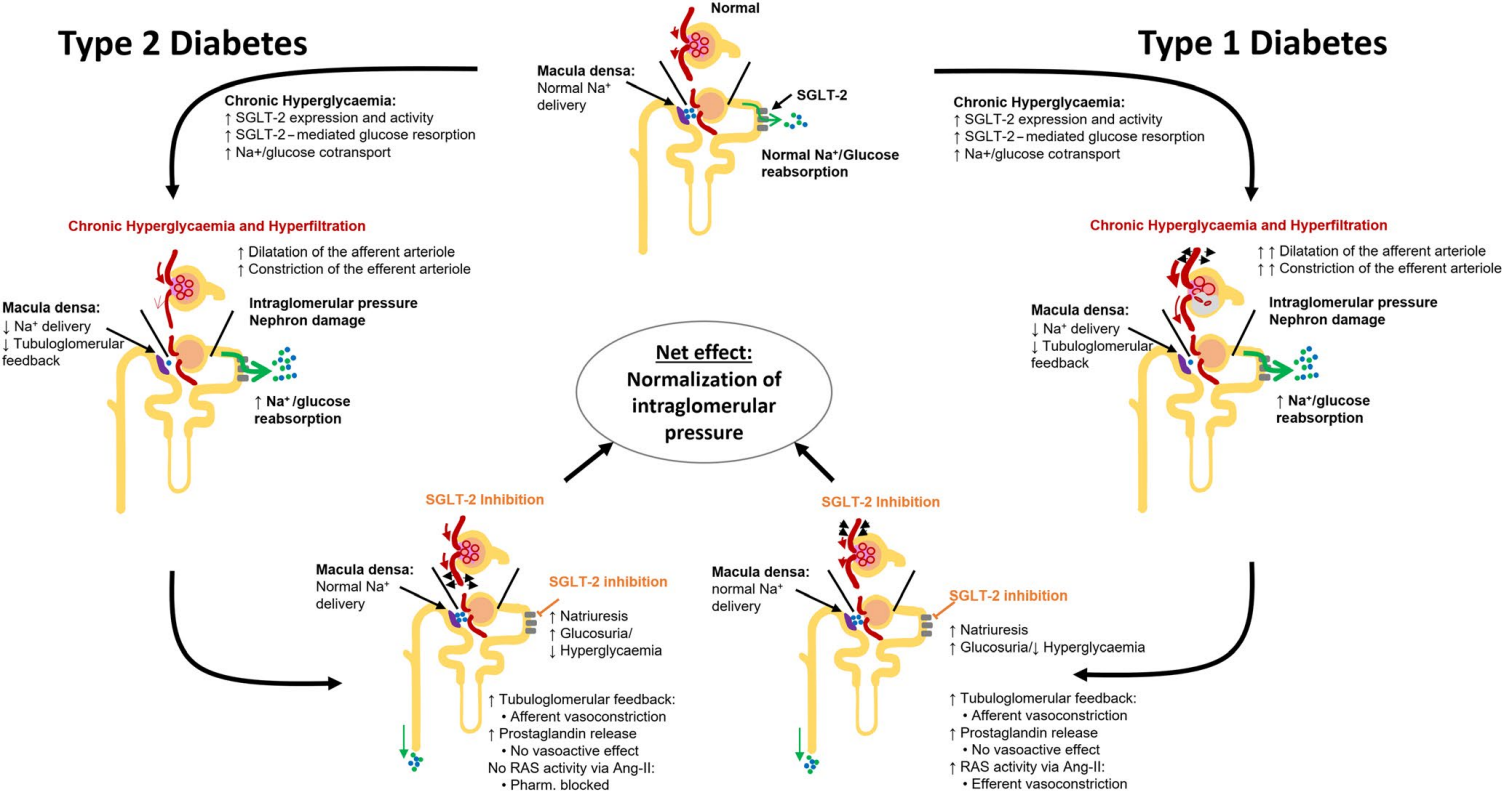
Suggested renoprotective mechanisms of SGLT‐2 inhibition in type 1 and type 2 diabetes after Heerspink, et al^18^ and Van Bommel et al^55^, respectively. Ang‐II, angiotensin‐II; RAS, renin‐angiotensin‐system; SGLT‐2, sodium/glucose co‐transporter 2

**Figure 2:**
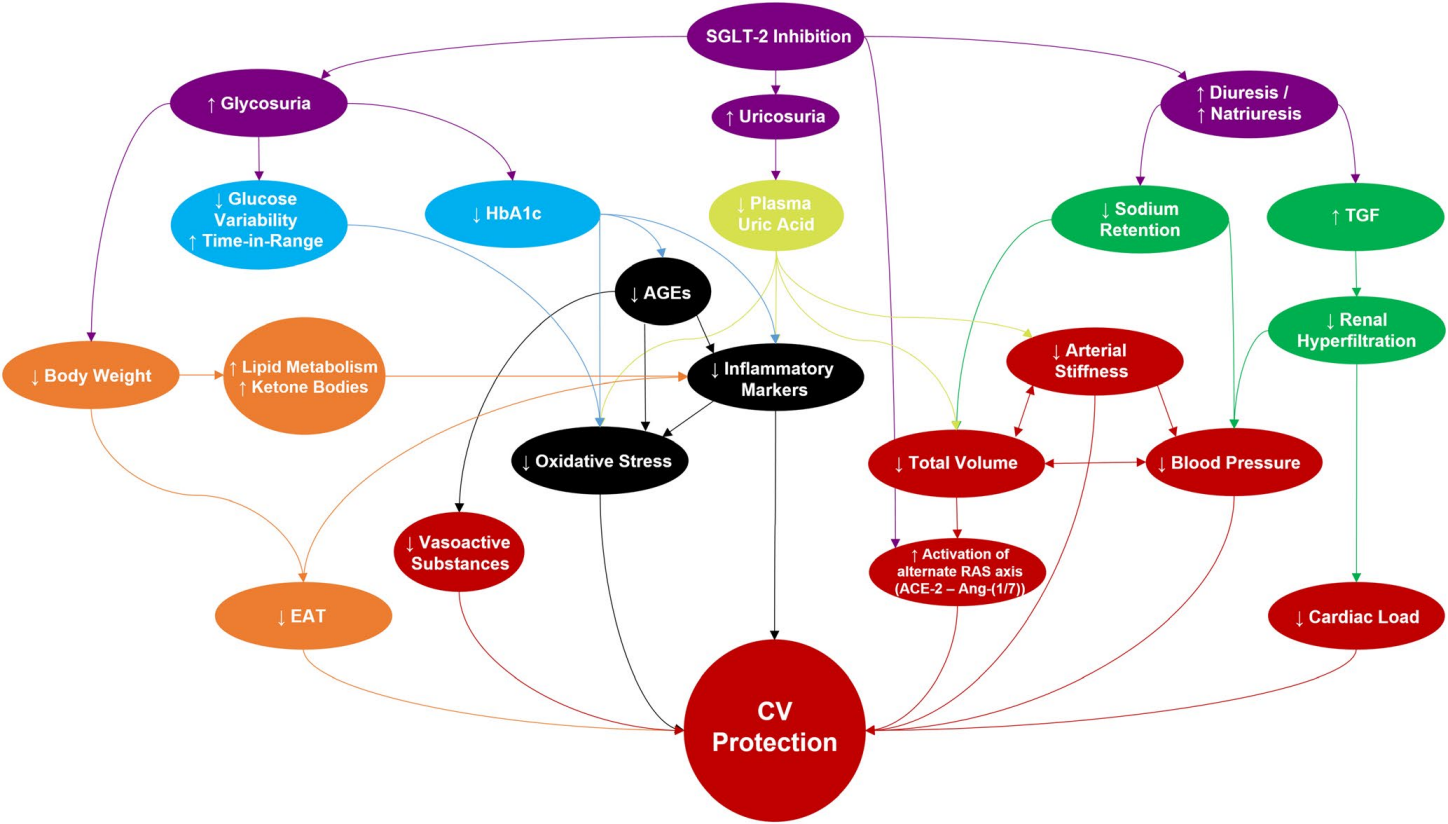
Summary of suggested cardioprotective mechanisms of SGLT‐2 inhibitors in type 1 and type 2 diabetes mellitus. ACE, angiotensin converting enzyme; Ang, angiotensin; EAT, epicardial adipose tissue; RAS, renin‐angiotensin‐system

As SGLT‐2–dependent glycaemic control is directly linked to the renal glomerular filtration rate (GFR), an attenuation of the glycaemic lowering effect with decreasing kidney function is implied.^15^ Yet, the CREDENCE trial demonstrated that also patients with T2DM and an estimated GFR (eGFR) of 30 to < 60 ml/min/1.73 m^2^ or a baseline urine albumin‐to‐creatinine ratio (UACR) >1000 mg/g benefit from SGLT‐2i treatment.^20^ This can most likely be attributed to other mechanisms of SGLT‐2is than just glycosuria. These effects are discussed throughout the manuscript, summarized in Figure 1 and Figure 2, and include (1) an increase in diuresis and natriuresis, resulting in restoration/preservation of the renal tubuloglomerular feedback and decreased sodium retention, positively impacting BP, (total) plasma volume, the renin‐angiotensin system (RAS) and cardiac load; (2) an increase in uricosuria, likely affecting downstream inflammatory markers, arterial stiffness and oxidative stress; and (3) downstream effects of glycosuria, such as caloric loss resulting in weight loss and metabolic shifts, as well as a reduction in other effects mediated by excess blood glucose like oxidative stress and pro‐inflammatory signalling. These mechanisms may be seen as base for comparison of benefits from T2DM to T1DM as they largely affect common clinical features of DM, albeit often of different genesis. These common clinical features encompass increased BP, body weight, dyslipidaemia, inflammatory signalling, cardiac and renal failure, and even IR in some cases.

## 3 UNMET MEDICAL NEED IN T1DM

Even though management of micro‐ and macrovascular comorbidities in T1DM has improved over the last 15 years, patients with T1DM still face a substantially increased risk of all‐cause mortality and of developing CVD like coronary heart disease (CHD)^21^ or heart failure (HF).^22^ An analysis of the Swedish National Diabetes Register revealed that patients with T1DM still suffer from a much higher standardized incidence rate for death from any cause, death from CVD, and hospitalization for CVD compared to matched controls, although significant decreases in levels of risk factors like high levels of low‐density lipoprotein (LDL)‐cholesterol, systolic BP (SBP) and prevalence of macroalbuminuria were observed in those patients between 1999 and 2014.^21^ However, an overall increase in mean HbA1c from 66.2 to 68.4mmol/mol [8.2% to 8.4%] was also observed in those patients from 1999 to 2014.^21^ These results are mirrored by other registries such as the Type 1 Diabetes Exchange Registry in the United States: the latest update (2016‐2018) suggests a small but noticeable increase in mean HbA1c in nearly all age groups except infants (2‐5 years old), compared to the initial enrolment population in 2010‐2012.^23^ It is important to recognize that all age groups had a mean HbA1c well above 7% [53 mmol/mol]. In the most recent update, the highest mean HbA1c was observed in the adolescent age group (13‐17 years, mean HbA1c 9.2% [77 mmol/mol]) and the lowest mean HbA1c in the oldest age group (≥50 years, mean HbA1c 7.7% [61 mmol/mol]).^23^

These developments become particularly critical in the light of known complications of inadequate glycaemic control: the Diabetes Control and Complications Trial (DCCT)/Epidemiology of Diabetes Interventions and Complications (EDIC) [DCCT‐EDIC] study demonstrated that intensive glycaemic control reduces the long‐term risk of developing macrovascular disease in T1DM.^24^ A more recent study confirmed that inadequate glycaemic control increases all‐cause mortality as well as death from CVD, yet also demonstrated that even a HbA1c ≤ 6.9% [52mmol/mol] leads to a nearly 3‐fold increased risk of dying from CVD in patients with T1DM, while a HbA1c ≥ 9.7% [83mmol/mol] was associated with an up to 10‐fold increased risk for death from CVD.^25^ A recent meta‐analysis on the relation of microvascular complications and the time in normoglycaemic range (time in range, TiR) in the DCCT study suggested that microvascular complications strongly increase depending on the time spent outside of normal glucose range (>70 to ≤ 180 mg/dL), with an up to 7‐fold risk of developing microalbuminuria and retinopathy with a TiR < 30%.^26^

Along this line, it is important to be aware of renal (dys‐)function in T1DM: it has been demonstrated that an increase in HbA1c (and diabetic hypertension, for which high HbA1c is a risk factor^27^) is an independent risk factor for the development of end‐stage renal disease (ESRD) in patients with T1DM.^28^ Micro‐ and macroalbuminuria as well as ESRD yet again elevate the risk for mortality and CVD such as coronary events and stroke in patients with T1DM.^29^, ^30^ Therefore, it is crucial to find the proper balance between tight glycaemic control to reduce micro‐ and macrovascular risk in clinical practice, and anticipation of hypoglycaemia, to increase overall life expectancy and quality of life of patients with T1DM.

## 4 OUTCOMES OF SGLT‐2is IN T1DM AND T2DM

CVOTs for T2DM were introduced in 2008, and every hitherto newly approved glucose‐lowering drug for the treatment of T2DM has undergone a CVOT to evaluate its CV safety, often also investigating renal end‐points. CVOTs of three SGLT‐2is were published, namely CANagliflozin cardiovascular Assessment Study (CANVAS—canagliflozin),^31^ Dapagliflozin Effect on CardiovascuLAR Events (DECLARE‐TIMI 58—dapagliflozin)^32^ and Empagliflozin Cardiovascular Outcome Event Trial in Type 2 Diabetes Mellitus Patients (EMPA‐REG OUTCOME—empagliflozin).^33^, ^34^ In addition, a large trial with a primary composite renal outcome, the Canagliflozin and Renal Outcomes in Type 2 Diabetes and Nephropathy (CREDENCE—canagliflozin) ^20^ was published recently.

All SGLT‐2i CVOTs either met their primary end‐point 3‐point major adverse cardiovascular events (3P‐MACE, met in CANVAS, EMPA‐REG) or showed a significant reduction in CV outcomes in co‐primary (DECLARE‐TIMI 58), secondary and exploratory end‐points. A meta‐analysis of the outcomes of CANVAS, EMPA‐REG OUTCOME and DECLARE‐TIMI 58 demonstrated an overall significant improvement of 3P‐MACE in patients with previous CVD (secondary prevention), yet not in patients with multiple risk factors (primary prevention).^35^ Similarly, the composite of hospitalization for heart failure (HHF) and CV death was significantly improved in patients in secondary prevention, yet not in primary prevention.^35^ However, in both primary prevention and secondary prevention, significant improvement of up to nearly 50% in the renal composite end‐point of renal worsening, ESRD, or renal death was revealed.^35^ It has to be noted that available data on the use of SGLT‐2is in patients with an eGFR < 60 ml/min/1.73 m^2^ are limited as the DECLARE‐TIMI 58 and EMPA‐REG OUTCOME trials included only 7.4% and 26% of patients with an eGFR < 60 ml/min/1.72m^2^, respectively,^33^, ^36^ and the CANVAS programme 22.8% of patients with an eGFR < 60 ml/min/1.72m^2^ ^37^ at baseline. Best currently available data are from the CREDENCE trial, with around 59.8% of the included patients presenting with an eGFR < 60 ml/min/1.73 m^2^ at baseline.^20^

The CREDENCE trial confirmed the secondary/exploratory renal outcomes reported in SGLT‐2i CVOTs, fortifying the idea of a class effect of SGLT‐2is, particularly in relation to renal benefits.^20^ In CREDENCE, canagliflozin demonstrated superiority compared to placebo in regard to the primary outcome (composite of ESRD, doubling of serum creatinine level from baseline, or death from renal or CV disease), the renal‐specific composite outcome (ESRD, doubling of serum creatinine level from baseline, or renal death), and occurrence of ESRD. Two additional trials with focus on kidney disease will be published in the next years to expand knowledge on renal outcomes of SGLT‐2is: DAPA‐CKD (dapagliflozin, trial population: T2DM and non‐DM; clinicaltrials.gov‐ID: NCT03036150) and EMPA‐KIDNEY (empagliflozin, trial population: T2DM, non‐DM and T1DM; clinicaltrials.gov‐ID: NCT03594110).

So far, there are no trials reporting CV or renal outcomes of SGLT‐2is as an adjunct therapy in T1DM. In contrast to CVOTs in T2DM, SGLT‐2i trials conducted in T1DM were considerably shorter, focused on efficacy and safety rather than CV or renal outcomes and had considerably smaller trial populations making them underpowered compared to CVOTs. However, these trials gave a first insight into the effects of SGLT‐2is on risk factor management in T1DM. So far, a phase II trial for canagliflozin^7^, ^8^ and several phase III clinical trials for dapagliflozin (Dapagliflozin Evaluation in Patients with Inadequately Controlled Type 1 Diabetes, DEPICT‐1 and −2^2^, ^3^, ^4^) and empagliflozin (Empagliflozin as Adjunctive to inSulin thErapy, EASE‐2 and −3^6^) and their effects as an adjunct treatment in T1DM were published. The canagliflozin trial demonstrated reductions in HbA1c, glycaemic variability, insulin dose, body weight and an increase in treatment satisfaction.^7^, ^8^ Similarly, both the DEPICT and EASE trial programmes showed a reduction in HbA1c, body weight, BP (particularly SBP), total insulin dose and glycaemic variability ^2^, ^3^, ^4^, ^6^ compared to placebo. Reduced rates of hypoglycaemic events were observed in the DEPICT and EASE trials, yet accompanied by increased rates of DKA.^3^, ^4^, ^6^ Of note, in the 26‐week EASE‐3 trial, the low dose of empagliflozin (2.5mg) demonstrated DKA events comparable to the placebo group (0.8% and 1.2%, respectively), yet with less pronounced glycaemic control (significant HbA1c reduction, no significant TiR improvements) compared to the higher doses of empagliflozin.^6^ However, when paralleled to the 24 week DEPICT‐1 trial, DKA events for the lower 5mg dose of dapagliflozin were also comparable to placebo (both 1%) and differed only after the 28‐week long‐term extension period of DEPICT‐1 (4.0% and 1.9%, respectively),^2^, ^3^ at which point DKA events were comparable between the 5mg and 10mg dose of dapagliflozin. This indicates that more, but especially longer trials are needed to investigate effects of low‐dose SGLT‐2 inhibition on DKA in T1DM. A recently published real‐world, secondary data analysis of off‐label use of SGLT‐2is as an adjunct therapy to insulin in T1DM in the United States from 2013 to 2018 revealed a real‐world DKA incidence in patients with T1DM of around 7.3 (5.81‐9.08) per 100 patient‐years.^38^ A meta‐analysis of controlled clinical trials with SGLT‐2is in T1DM reported a risk ratio (RR) between 2.90 and 3.11, depending on low or high dose, respectively.^39^

## 5 DKD AND RENAL PROTECTION—COMMONALITIES, DIFFERENCES, AND TRANSFERABILITY IN T1DM AND T2DM

Clinically, DKD is defined as persisting, severely elevated albuminuria of > 300 mg/24 h or an albumin‐to‐creatinine ratio (ACR) of > 300 mg/g, usually accompanied by a progressive loss of GFR, concomitant diabetic retinopathy and characterized by lack of other forms of kidney disease.^40^, ^41^ DKD occurs in 20%‐40% of all DM patients (ca. 25%‐30% in T1DM and ca. 5%‐40% in T2DM), accounting for a major cause of mortality in DM.^42^, ^43^ Risk factors for DKD are manifold and include (1.) susceptibility factors like age, race/ethnicity, sex and genetic risk factors; (2.) the initiation factor hyperglycaemia; and (3.) progression factors like hypertension, obesity^44^ or lipids.^45^ The development of DKD has been best characterized for T1DM; however, the clinical and histological presentation of DKD is similar in T1DM and T2DM^46^ (Table 1).

**Table 1:** Characteristics of diabetic kidney disease in type 1 and type 2 diabetes mellitus

Characteristics of DKD	Type 1 diabetes mellitus	Type 2 diabetes mellitus
Estimated amount of patients with DKD	25%‐30%^43^ (estimations of over 75% also exist^129^)	5%‐40%^42^
Risk factors^44^	(1) Susceptibility factors: age, race, ethnicity, sex, genetics	(1) Susceptibility factors: age, race, ethnicity, sex, genetics
	(2) Initiation factors: hyperglycaemia	(2) Initiation factors: hyperglycaemia
	(3) Progression factors: hypertension, endothelial dysfunction, inflammation *(obesity)*	(3) Progression factors: hypertension, obesity, lipids, endothelial dysfunction, inflammation
Hyperfiltration and progressive loss of GFR^48^	Yes	Yes
Presence of albuminuria	Yes 2%‐10% nonalbuminuric DKD^49^, ^50^	Yes, but: misdiagnosed as DKD in up to 50% of patients with severe hypertension, rapid loss of eGFR and macroalbuminuria (nondiabetic CKD)^46^
Pathophysiology^44^, ^47^	Renal hypertrophy, renal fibrosis, hemodynamic changes, glomerulosclerosis, inflammation, ischaemia, overactivation of RAS	
Clinical and histological characteristics^129^	Similar and progressive Histological: Diabetic glomerulosclerosis; widening of glomerular basement membrane; mesangial matrix accumulation; podocyte foot process widening and effacement; loss of podocytes; alterations in glycosaminoglycans of the glomerular filtration barrier; Clinical: Patients with DKD more likely to be male and smokers with poor glycaemic control, high blood pressure, *insulin resistance*, dyslipidaemia (high triglycerides, low‐HDL cholesterol) and endothelial dysfunction Increase in risk for ESRD and comorbidities with increase in albuminuria; Parallel progression of other common comorbidities such as retinopathy or CVD; CVD also particularly linked to loss of GFR Rising BP (yet not in ‘hypertensive range’)	

Pathophysiology of DKD in both T1DM and T2DM has been associated with a variety of processes: major structural changes during the progression of DKD include endothelial fenestrations, mesangial expansion, loss of podocytes with effacement of foot processes and glomerular sclerosis.^44^, ^47^ Hyperglycaemia, as well as IR in T2DM, plays a major role in the later stages of DKD by inducing renal haemodynamic changes, ischaemia, inflammation and overactivation of the RAS, all of which lead to macroalbuminuria, glomerulosclerosis, tubulointerstitial fibrosis and ultimately renal fibrosis and ESRD.^47^ Important and tightly interwoven factors for haemodynamic changes and pro‐inflammatory signalling in the kidneys are endothelin‐1 (ET‐1), prostaglandin, vascular endothelial growth factor (VEGF), reactive oxygen species (ROS) and nitric oxide (NO).

### 5.1 Hyperfiltration, glycaemic control and SGLT‐2is in DKD

The major commonality of T1DM and T2DM—hyperglycaemia—was shown to likely be the biggest risk factor in the initiation and progression of hyperfiltration. Hyperfiltration as first step of DKD is experienced by 10%‐67% of patients with T1DM and 6%‐73% of patients with T2DM.^48^ During hyperfiltration, GFR increases of up to 27% in T1DM and 16% in T2DM patients have been observed.^48^ Nephrons usually are predisposed to progressive damage by hyperfiltration before albuminuria and other DKD pathologies manifest.^48^ Subsequent to the development of single‐nephron hyperfiltration is progression to whole‐kidney hyperfiltration, as with increasing amounts of damaged glomeruli, remaining healthy nephrons undergo functional and structural hypertrophy in order to maintain kidney function. This was supported by longitudinal studies which demonstrated that in patients with T1DM and T2DM and whole‐kidney hyperfiltration, GFR declined more rapidly, compared to patients with normal whole‐kidney GFR at baseline.^48^ Of note, a recent study reported around 2% of T1DM patients had nonalbuminuric DKD at baseline, associated with an increased risk of CVD and all‐cause mortality.^49^ Another study reported that up to 10% of patients with T1DM manifest with continuously declining GFR in the absence of macroalbuminuria.^46^, ^50^ Thus, declining GFR and albuminuria cannot be considered as sequential steps of DKD, but rather as two actively deteriorating conditions with different basis, each individually contributing to the development of DKD.^46^, ^47^, ^51^, ^52^

Concomitant to an increase in glucose reabsorption by SGLT‐2 is a decrease in the delivery of sodium chloride (NaCl) to the macula densa.^18^ Upon decrease in NaCl delivery to the macula densa, less adenosine, a strong vasoconstrictor, is produced by the macula densa cells during the energy‐consuming process of sodium export. The decrease in adenosine in turn decreases the tubuloglomerular feedback, dilating the afferent arteriole, thus paving the way for renal hyperperfusion and glomerular hyperfiltration.^18^ Upon SGLT‐2 inhibition, increased NaCl delivery to the macula densa restores the tubuloglomerular feedback, supporting afferent arteriole constriction, effectively decreasing intraglomerular pressure and GFR and promoting normal glomerular perfusion and filtration.^18^, ^53^ This suggests that not the reduction in blood glucose but rather the decreased reuptake of NaCl in the distal tubule is responsible for the beneficial effects. In a cohort of T1DM patients, both patient groups with either hyper‐ or normofiltration had reductions in mean HbA1c, yet only the hyperfiltration group showed a reduction in inulin‐derived measured GFR (mGFR), renal blood flow and renal vascular resistance, promoting the concept of direct intrarenal effects.^18^, ^54^

A recent study investigated the effects of dapagliflozin on renal function in a small cohort of patients with T2DM (n = 24), compared to the sulfonylurea gliclazide.^55^ The authors demonstrated that dapagliflozin lowered the mGFR independent of glucose control on top of standard of care (RAS blockade, metformin). However, they observed different mechanisms in patients with T2DM than for patients with T1DM and hyperfiltration: instead of the above described afferent vasoconstriction through a restoration of the tubuloglomerular feedback, they observed efferent vasodilation and no change in renal vascular resistance. They reasoned that this variance may be due to age of participants and course and treatment of T2DM—while described effects of afferent vasoconstriction may well apply to a younger T1DM population, the majority of the T2DM population is much older and already receives long‐term RAS inhibitor treatment. The authors propose that the potential vasoconstrictive action of an increase in adenosine, resulting from an increase in tubuloglomerular feedback, may be countered by increased prostaglandin synthesis—inducing efferent vasodilation, particularly while Ang‐II‐action is pharmacologically inhibited. In addition, the authors reason that an increase in adenosine may directly contribute to efferent vasodilation, as afferent vasoconstriction may already be at its limits in T2DM. Overall, this effect of SGLT‐2is may result in an unchanged preglomerular tone but a net reduction in postglomerular tone, thereby reducing the mGFR.^55^ However, the net effect on progression of renal disease in DM may still be comparable: normalization of intraglomerular pressure contributes to the observed improvements, as increased intraglomerular pressure and glomerular wall tension have been linked to renal fibrosis and inflammation in animal models.^18^

Recent studies demonstrated that SGLT‐2is may not only have a positive impact on the development and progression of hyperfiltration, but may also exert positive effects in patients already presenting with albuminuria and declining GFR. For example, an analysis of the DECLARE‐TIMI 58 trial (T2DM) demonstrated that dapagliflozin had significant positive effects on the renal‐specific outcome in patients with an eGFR ≥ 90 ml/min/1.73m^2^ and 60 to < 90 ml/min/1.73m^2^ and close to significant positive effects in patients with an eGFR < 60 ml/min/1.73m^2^ (*P* = .059, likely not significant due to small patient numbers). Similarly, dapagliflozin significantly reduced the risk for the renal‐specific outcome across all ranges of UACR (<30 mg/g to > 300 mg/g).^36^ Correspondingly, for T1DM, a post hoc analysis of the DEPICT‐1/‐2 trials in patients with existing albuminuria at baseline demonstrated that application of dapagliflozin (10 mg) as an adjunct therapy to insulin resulted in a significantly reduced percent change in UACR.^56^

### 5.2 Impact on glomerular hypertension

It has been demonstrated that particularly an early decline in the eGFR slope is correlated with subsequent risk of ESRD, for which reason maintaining GFR is the primary focus for prevention and slowing the progression of DKD to ESRD.^46^ Hyperglycaemia activates the RAS ^57^ and high local concentrations of angiotensin II at the efferent arteriole result in vasoconstriction which, combined with a reduction in tubuloglomerular feedback, effectively increases intraglomerular pressure (intraglomerular hypertension) and glomerular hyperfiltration.^44^, ^58^

Thus, current first‐line treatment for hypertension and DKD in DM are RAS blockers. Lowering systemic BP was shown to slow the initiation and progression of DKD in T1DM ^59^ and T2DM.^60^ However, RAS blockers seem to be most effective in patients with high levels of albuminuria and do not halt the progression of DKD.^46^ In addition, RAS blockers often are contraindicated in patients with advanced ESRD and termination of RAS blockade may become indispensable in patients with stage 4 or 5 kidney disease due to excessive lowering of eGFR as well as hyperkalaemia.^46^ Furthermore, angiotensin II–receptor blockers (ARBs) are unable to prevent the development of DKD in T2DM^46^ and T1DM, as demonstrated by the Renin Angiotensin System Study (RASS) of adults with uncomplicated T1DM^61^ and the Adolescent Cardiorenal Intervention Trial (AdDIT).^62^ These drawbacks of RAS blockade have been suggested to be connected with the inability of RAS inhibitors to facilitate glycaemic control.^46^ In this regard, SGLT‐2is may offer an opportunity, possibly in the prevention but particularly in the early stages of DKD, as they not only improve glycaemic control but also play a role in the maintenance of GFR and nephroprotection by mediating intraglomerular effects through indirect mechanisms like BP reduction and direct mechanisms as described in the previous section.

More studies for primary prevention of DKD with SGLT‐2is are needed in T1DM and T2DM as reliable data are lacking so far. A recent study demonstrated a promotion of global RAS activation upon SGLT‐2i treatment in T1DM.^63^ It was observed that 8‐week empagliflozin treatment increased levels of angiotensin I and angiotensin II, as well as levels of Ang‐(1‐5), reflecting a conversion of Ang‐(1‐7) to Ang‐(1‐5) and decreased plasma angiotensin converting enzyme (ACE) activity. The authors reasoned that SGLT‐2is may induce favourable ACE‐2 levels and that a combination of SGLT‐2i and ACE inhibition may synergistically and favourably boost the alternative RAS axis, resulting in favourable RAS profiles which may decrease the risk of DKD in patients with DM.^63^ For EMPA‐REG OUTCOME, in which 81% of the patients received ACE inhibition or ARBs, it has been speculated that ACE inhibition and ARBs in combination with SGLT‐2 inhibition lead to an enhancement of the angiotensin pathway from Ang‐(1‐10) to Ang‐(1‐7) and thus cardioprotective effects.^64^ These aspects likely also are reflected in the results from a subanalysis of the DECLARE‐TIMI 58 trial: patients with baseline ACE inhibitor or ARB use showed a significant reduction of 50% of the composite renal‐specific outcome (sustained decrease in eGFR by at least 40% to less than 60 ml/min/1.73m^2^; ESDR, or renal death), while patients without baseline use of ACE inhibitors or ARBs showed a 23% reduction in the composite renal‐specific outcome (not significant).^36^ In an exploratory analysis of EMPA‐REG OUTCOME,^65^ the difference in eGFR between empagliflozin and placebo groups was more pronounced in patients with macroalbuminuria at baseline. This suggests that even in patients at high risk of DKD progression, SGLT‐2is are able to reduce eGFR decline up to 75%, occurring in addition to a coexisting (already nephroprotective) RAS blockade. Caution needs to be taken with SGLT‐2i treatment in later stages of DKD, as to whether eGFR levels are still in adequate ranges for SGLT‐2i treatment,^66^ and more studies are required for patients with ESRD who were often excluded in relevant trials (eg in CREDENCE, patients with an eGFR < 30 ml/min/1.73m^2^ were excluded^20^).

### 5.3 Impact of SGLT‐2 is on renal hypoxia

Compared to placebo, all SGLT‐2 is (canagliflozin, dapagliflozin and empagliflozin) have demonstrated a modest increase in haematocrit which cannot be explained solely by their diuretic effect.^67^ A current theory revolves around reduction in metabolic stress in the kidney by SGLT‐2is: metabolic stress (increased energy and oxygen demand) due to hyperglycaemia in DM may cause erythropoietin‐producing fibroblasts near the proximal tubules in the kidney to transform into myofibroblasts which produce fibrogenic molecules, resulting in decreased serum erythropoietin levels, positively correlating with increasing HbA1c.^67^ By diminution of hyperglycaemia and restoration of the tubuloglomerular feedback, energy and oxygen demand and renal metabolic stress are reduced and myofibroblasts are proposed to revert back to erythropoietin‐producing fibroblasts, effectively increasing serum erythropoietin and haemoglobin, augmenting oxygen delivery to renal as well as cardiac cells.^67^ Another study showed that treatment of patients with T2DM with dapagliflozin resulted in a significant reduction in hepcidin which, in turn, has been found increased in pro‐inflammatory states and is a known suppressor of erythropoiesis,^68^ providing another plausible mechanism of increased haematocrit and renoprotection.

## 6 CVD AND CARDIOVASCULAR PROTECTION—COMMONALITIES, DIFFERENCES, AND TRANSFERABILITY IN T1DM AND T2DM

DM significantly increases the risk for CVD and CVD is the leading cause of mortality and morbidity in T1DM and T2DM.^69^ Mechanisms of development of CVD in T1DM and/or T2DM are not entirely clear yet, for the main reason that they are manifold. DM, IR, and obesity among other risk factors like lifestyle, smoking habits, and age are responsible for pathological changes like hyperglycaemia, dyslipidaemia, hypertension, and an increase in inflammatory processes. A large meta‐analysis showed that T2DM confers an about 2‐fold higher risk for CHD and other vascular deaths^70^ and CVD mortality risk in T2DM ranges from 1.5‐fold to 4.6‐fold.^71^ The prevalence of CVD in T1DM varies considerably depending on duration of DM.^41^ T1DM confers a 2‐fold to 4‐fold increased risk of subclinical coronary artery disease (CAD) and is associated with an up to 20‐fold and 40‐fold increased risk for ischaemic heart disease in young men and women (under the age of 40), respectively, compared to nondiabetic adults,^71^ thus usually occurring at much younger age, hence a greater relative risk.^41^, ^71^

One of the most important risk factor linking both diseases is chronic hyperglycaemia which serves as both initiation and progression factor, affecting several downstream mechanisms.^72^ A Finnish study, following up patients with DM for 18 years, showed that CV and total mortality stratified to glycaemic control were increased in controlled DM (HbA1c ≈8% [64mmol/mol]) in patients with T2DM, compared to matched patients with T1DM, yet aligned with increasing HbA1c resulting in nearly equal deaths per 1000 person‐years in uncontrolled DM (HbA1c > 11% [97mmol/mol]) for T1DM and T2DM.^72^ For both, T1DM and T2DM, it has been shown that intensive glycaemic control reduces the risk of CVD.^71^ So far, and in contrast to T2DM, tight glycaemic control in T1DM is facilitated by intensified insulin regimens only, often associated with side effects like weight gain. In this light and the increasing prevalence of obesity and often concomitant IR in patients with T1DM,^73^, ^74^ many of the risk factors for CVD (eg obesity, dyslipidaemia and hypertension) become increasingly prominent in both, T1DM and T2DM.

### 6.1 SGLT‐2 inhibition and the impact of glycaemic control on CVD

Hyperglycaemia has been proposed to play a role in chronic low‐grade inflammatory processes, thereby damaging endothelial integrity, to affect BP by various mechanisms like promoting oxidative stress by increasing advanced glycosylation end‐products (AGEs) and reactive oxygen species (ROS) production, to influence vasoactive substances, and many other mechanisms ultimately promoting CVD. While glycaemic control may play an important role in lifelong risk reduction, it has been argued for the setting of CVOTs in T2DM that cardioprotective effects are independent of glycaemic improvements for two reasons: first, the changes observed in the placebo subtracted difference in glycaemic control in SGLT‐2i CVOTs were modest and in similar ranges as in dipeptidyl‐peptidase‐4 inhibitor (DPP‐4i) CVOTs which all showed neutral effects on CV outcomes.^75^ Second, in the CVOTs which showed superiority with respect to 3P‐MACE (EMPA‐REG OUTCOME and CANVAS), Kaplan‐Meier curves start to drift apart as early as 6 to 12 months into the study. Similar effects are observed in DECLARE‐TIMI 58 when considering the co‐primary outcome (cumulative incidence for CV death and HHF), suggestive of a more prompt mechanism than glycaemic control.^31^, ^32^, ^33^, ^75^ However, due to different pathologies, patients with T1DM are exposed to inadequate glycaemic control much earlier and longer in life than patients with T2DM. Tight glycaemic control was shown to be highly beneficial for CV protection in T1DM^24^ and the final role of risk factor management for the treatment of CV comorbidities in T1DM needs to be further investigated.

### 6.2 Implications for the (diabetic) heart

All SGLT‐2 inhibitors showed remarkable and similar improvements of HHF in their corresponding CVOTs in T2DM, ranging from 33%^31^ to 39%^20^ (canagliflozin) over 27% (dapagliflozin)^32^ to 35% (empagliflozin)^33^ improvement of HHF and across the spectrum of renal disease with eGFRs from > 30 ml/min/1.73m^2^ to > 90 ml/min/1.73 m^2^.^76^, ^77^ In addition, treatment efficacy was demonstrated in both the primary and secondary prevention cohorts of the CANVAS and DECLARE programmes.^31^, ^32^, ^78^

Suggested mechanisms of SGLT‐2i–mediated benefits in the improvement of HF were recently reviewed elsewhere^77^ and include (1) improvement in ventricular loading conditions by a reduced preload (as an effect of natriuresis and osmotic diuresis) and reduced afterload (as an effect of reduction in BP and improvement in vascular function); (2) improved cardiac metabolism and bioenergetics; (3) inhibition of the sodium (Na^+^)/hydrogen (H^+^) exchange in cardiomyocytes (mediated by a proposed direct inhibition of the sodium/hydrogen‐exchanger‐1 by SGLT‐2is, decreasing intracellular sodium and calcium levels while increasing mitochondrial calcium ^79^); (4) reduced necrosis and fibrosis; and (5.) alterations in epicardial adipose tissue (EAT) mass and inflammatory signalling.

Consistently, a recent trial with 53 patients with T2DM and stable HF demonstrated that 6‐month treatment with dapagliflozin significantly decreased E/e’, left atrial volume index and left ventricular mass index, suggesting improvement in diastolic function, one of the presumed underlying pathologies of HF.^80^ Again, there is an interplay of the cardiorenal system: patients with DM and glomerular hyperfiltration were shown to have an elevated renal blood flow of up to 60%, compared to individuals with normofiltration.^48^ Renal blood flow represents around 25% of the cardiac output, and SGLT‐2is have shown to decrease renal blood flow by around 30% in hyperfiltrating patients with T1DM, which may translate into an approx. 8% decrease in cardiac output and thus act cardioprotective.^53^, ^54^ Particularly in the light of HF, it is remarkable that the benefits mediated by SGLT‐2 inhibition seem rather preserved, also in populations without diabetes: results from the DAPA‐HF trial showed that dapagliflozin, added on top of standard of care in patients with and without T2DM with HF with reduced ejection fraction (HFrEF), was able to significantly reduce CV death and prevent worsening of HF overall and in both groups (DM and non‐DM), compared to placebo.^81^

### 6.3 Impact on substrate utilization of the (diabetic) heart

It has been reported that SGLT‐2is promote the use of fatty acids by beta‐oxidation^82^ and ketone body oxidation,^83^ owing to the glycosuric effect of SGLT‐2is, resulting in carbohydrate‐dependent caloric loss. It was revealed in animal models that the decline of cardiac function in diabetes is due to a reduction in cardiac energy production caused by a combined decrease in fatty acid and glucose oxidation rates.^83^, ^84^ According to the ‘thrifty substrate’ hypothesis, increased ketone levels may present an efficient source for the energy generation of the heart, with up to 70% of its energy generated by beta‐oxidation of fatty acids if available, as an energetically more favourable way compared to glucose oxidation.^85^, ^86^ Also, it has been observed for empagliflozin in animal models that SGLT‐2is shifted myocardial metabolism away from glucose towards a energetically more favourable ketone body metabolism within 2 months.^87^ A more recent study, however, reported that in animal models of the failing diabetic heart also ketone oxidation was not altered.^83^ Importantly, in this model, empagliflozin rather increased overall cardiac energy production by an increase in glucose and fatty acid oxidation.^83^ The authors reason that it may be plausible that an empagliflozin‐dependent increase in serum ketone bodies may serve as additional substrate for cardiac energy production, while it per‐se does not alter ketone oxidation rates.^83^ Another approach was an untargeted metabolomics analysis of empagliflozin‐treated patients with T2DM and CVD: this suggested that also increased branched‐chain amino acid catabolism, caused by empagliflozin treatment, may improve the energy supply of the heart.^88^

### 6.4 Impact on hypertension

Hypertension is common among patients with DM, with a prevalence from 10% to 30% in T1DM, growing up to 67% after 30 years of T1DM in DCCT/EDIC,^89^ and around 60% in T2DM.^90^ In a recent large study, 35% of patients with T1DM and 41% of patients with T2DM presented with hypertension at baseline.^91^ Onset of hypertension is influenced by different factors in T1DM and T2DM: while hypertension usually develops years after the onset of T1DM, it is commonly already present at the time of diagnosis of T2DM and associated with obesity and IR.^90^, ^92^ In T1DM, atypical BP variations usually stem from the development of DKD, obesity, or hyperglycaemia (in the long run), and are often associated with chronic dysregulation of the RAS.^41^, ^92^ In DCCT‐EDIC, intensive glycaemic control was associated with reduced long‐term risk of nephropathy and hypertension.^93^

Decreases of up to ≈5mmHg in SBP ^33^ and up to ≈3mmHg in diastolic BP (DBP) ^32^ have been observed in CVOTs investigating SGLT‐2is. Similar diminutions of up to ≈5/≈2mmHg SBP/DBP were reported with SGLT‐2is in T1DM.^2^, ^3^, ^4^, ^6^ BP reduction likely is mediated by a combination of modest weight loss, modest glucose‐based osmotic diuresis and a small natriuretic effect and is comparable to BP‐lowering effects of established substances with predicted cardioprotective effects.^12^ Similar BP‐lowering effects of SGLT‐2is, as in T2DM, have been observed in T1DM; thus, transferability of these benefits from T2DM to T1DM, to at least some extent, seems reasonable.

Next to direct effects on BP, other effects likely contributing to BP reduction and CV safety are a small reduction in plasma uric acid concentration (shown in patients with T2DM) ^33^ and a decline in arterial stiffness (reported in patients with T1DM).^94^ In T1DM, elevated plasma concentrations of uric acid have been associated with onset and progression of DKD, including metabolic (IR and hyperglycaemia), CV (hypertension, endothelial dysfunction, and arterial stiffness) and kidney function abnormalities.^95^ Furthermore, plasma uric acid was associated with pro‐inflammatory signalling and activation of the RAS.^95^ In EMPA‐REG OUTCOME, reduced plasma uric acid was observed in patients treated with empagliflozin.^33^ The proposed underlying mechanism is an increased secretion of uric acid in exchange for glucose reabsorption via the GLUT9 transporter as result of increased glycosuria, natriuresis, and uricosuria due to the SGLT‐2i action, causing an overall decrease in plasma uric levels by 10‐15%.^18^ Plasma uric acid concentration has been shown to correlate positively with SBP and negatively with GFR and effective renal plasma flow in T1DM, yet was generally lower in patients with T1DM compared to healthy controls.^96^ Therefore, precise clinical relevance is still unclear, particularly in T1DM, but a positive cardio‐ and renoprotective effect cannot be excluded.^75^ Also, it has to be mentioned that recent clinical trials specifically investigating the CV effects of urate‐lowering therapies in patients with gout observed higher mortality in patients achieving lower serum urate.^53^, ^97^

Arterial stiffness and sodium retention, resulting in volume expansion, are other proposed mechanisms of hypertension in DM,^98^ and augmentation of arterial stiffness has been independently linked to negative CV, renal and retinal outcomes in T1DM.^99^ Hence, reduction in arterial stiffness on the one hand and decline of (total) plasma volume as consequence of diuretic effects of SGLT‐2is on the other hand present additional mechanisms by which SGLT‐2is may mediate protective effects for the CV system independent of the type of diabetes.^94^, ^98^

### 6.5 The link to obesity, dyslipidaemia and inflammation

Particularly T2DM, overweight, and obesity are tightly interwoven. However, the proportion of overweight/obese patients has increased dramatically also in T1DM,^41^, ^73^ likely related to epidemiological shifts in the overall population and tighter glucose control often coupled with more frequent and/or greater caloric intake (eg to avoid actual or perceived hypoglycaemia).^41^ While it is not entirely clear how overweight/obesity really impacts T1DM, it likely increases CVD risk factors such as visceral adiposity, BP, dyslipidaemia, and IR, as true for the nondiabetic population.^41^ It has been suggested that IR is not related to the contemporary level of glycaemic control in T1DM,^100^ yet the EURODIAB Study proposed that IR‐related risk factors predict CHD events in patients with T1DM and that IR may explain some lipid abnormalities in young patients with T1DM.^41^, ^101^, ^102^ A cross‐sectional analysis of CVD risk factors in the EURODIAB study demonstrated that patients with T1DM and CVD had increased triglycerides and decreased high‐density lipoprotein (HDL)‐cholesterol, compared to those without CVD.^71^, ^103^ Bad glycaemic control, higher body weight, and subsequent increases in IR have been linked to a more atherogenic cholesterol distribution in T1DM.^104^, ^105^, ^106^ The SEARCH study observed higher lipid values in youths with T1DM compared to those without T1DM in relation to glycaemic control.^107^ These observations are matched by a recent study which reported that the use of lipid‐lowering therapy in patients with T1DM significantly improved all‐cause death as well as CV death and stroke.^108^

SGLT‐2is caused a loss of mean total body weight of up to ≈2kg in T2DM CVOTs ^31^, ^32^, ^33^ and between ≈2kg and ≈4kg in T1DM.^2^, ^3^, ^4^, ^6^, ^7^ These small weight changes are unlikely to directly mediate the cardio‐ or renoprotective effects of SGLT‐2is.^75^ On the contrary, it has been proposed that SGLT‐2i monotherapy is insufficient for the treatment of obesity.^109^ There are first indications of how SGLT‐2is may aid the treatment of IR, obesity, and associated low‐grade inflammation: first, glycosuria results in carbohydrate‐related calorie loss, partially responsible for the observed weight losses.^110^ This likely causes the SGLT‐2–mediated shift of substrate utilization to beta‐oxidation (fatty acid oxidation), further contributing to weight loss.^82^ In addition, there are first hints of a SGLT‐2i‐mediated ‘browning’ of white adipose tissue into brown adipose tissue by increasing expression/activity of uncoupling protein 1, resulting in increased thermogenesis and increased energy expenditure.^111^

CVD development in DM and obesity is linked by the presence of low‐grade inflammation among other factors.^112^ Obesity and T2DM are linked by a low‐grade systemic inflammation. These cytokines are thought to contribute to systemic inflammation and lipid accumulation, resulting in the development of endothelial dysfunction and subsequent CVD.^92^ In T1DM, inflammation clearly represents a crucial factor in the pathology and there is a considerable overlap of inflammatory factors and processes in T1DM and T2DM.^41^, ^113^, ^114^, ^115^ Animal models gave indications that SGLT‐2is may improve inflammatory signalling particularly in the liver and kidney, as well as improving IR by reducing inflammatory signalling, resulting in reduced macrophage recruitment and M1 polarization but increased M2 polarization.^111^ The M2 polarization of macrophages was suggested to convey anti‐inflammatory signalling in adipose tissue, to benefit insulin sensitivity and to detain the progression of IR.^111^ A direct connection of SGLT‐2is, adipose tissue, inflammation and the heart has also been proposed: a study investigating the effect of dapagliflozin on EAT mass in T2DM reported that the dapagliflozin group showed a significantly greater decrease in EAT compared to the conventional treatment group, correlating to reductions in body weight. In addition, a significantly larger decrease in inflammatory signalling correlating to changes in EAT was observed.^116^ The authors concluded that dapagliflozin might on the one hand improve systemic metabolic and inflammatory parameters, and on the other hand decrease EAT volume, thereby decreasing CV risk.^116^

In summary, the hypothesis of reduced inflammation is based on several observations: (1.) reduction in body weight, known to contribute to the decline of low‐grade inflammation in obese individuals; (2.) reduction in hyperglycaemia, shown to reduce inflammatory markers; (3) reduction in serum uric acid, a known activator of the NALP3 inflammasome; (4.) increase in ketone bodies conveying anti‐inflammatory signalling; (5.) reduction in oxidative stress; and (6.) the suppression of the pro‐inflammatory AGE‐RAGE‐axis, as reviewed elsewhere.^117^, ^118^ In future, effects on body weight in T1DM may receive more attention as simultaneous appearance of T1DM and metabolic syndrome or even ‘double diabetes’ (IR in patients with T1DM) slowly become more prevalent. A recent study reported as much as 25.5% of patients with T1DM to also present with the metabolic syndrome (defined as obesity, hypertension, and/or dyslipidaemia),^119^ and the Swedish National Diabetes Registry noted a substantial increase in overweight/obese patients with T1DM.^120^

### 6.6 Modulation of the sympathetic nervous system

Studies suggest that CV benefits of SGLT‐2is in T2DM are not solely mediated by direct effects (increased diuresis, natriuresis, uricosuria, and glycosuria and associated mechanisms), but also through modulation of the sympathetic nervous system (SNS).^121^ Chronic overactivation of the SNS is known for several (chronic) diseases such as the metabolic syndrome, obesity, hypertension, HF, kidney disease and T2DM.^121^ Over‐activity of the SNS has been postulated to be involved in endothelial dysfunction and arterial stiffness, but seems to centralize around the kidney in T2DM, with renal stress stimulating the sympathetic centre.^121^ The mechanism of impact of SGLT‐2is on the SNS and its activation is still unclear—a current hypothesis circles around the higher energy consumption of the Na^+^/K^+^ ATPase of the proximal tubular cells of the kidneys upon an increase in glucose uptake and the concomitant increase in sodium uptake in the diabetic state, which results in a tissue‐specific local decrease in oxygen partial pressure, ultimately promoting a progress of interstitial fibrosis.^121^ Animal models showed that empagliflozin effectively reduced the low frequency of SBP in OLETF rats which were given high‐salt drinking water ^122^ and that dapagliflozin reduced renal and cardiac sympathetic activity in mice on a high‐fat diet.^123^ In patients with T2DM, it was demonstrated that empagliflozin abolished sympathetic activation as early as four days into treatment.^124^ However, it is yet unclear if these effects can be transferred from T2DM to T1DM, as the autoimmune nature of T1DM may also affect the SNS, as recently shown for SNS pancreatic innervation.^125^ More studies are needed to shed light on the involvement of the autonomous nervous system in T1DM and the potential effects SGLT‐2is may exert on it.

## 7 CONCLUSION AND CLINICAL IMPLICATIONS

Despite medical and technological advancements, there still is a major unmet need in the treatment of T1DM. Patients with T1DM are faced with increased rates of CV and all‐cause mortality, CVD and renal disease. SGLT‐2is have shown to be a promising treatment option in T2DM, demonstrating strong CV and renal benefits in designated CVOTs. As SGLT‐2is have insulin‐independent mechanisms of action, the question of transferability of these effects from T2DM to T1DM is debated heatedly. First results from trials with SGLT‐2is as adjunct therapy in T1DM (DEPICT‐1/‐2; EASE‐2/ −3) have demonstrated effects on CV risk factors like HbA1c, BP, and body weight in T1DM, comparable to results obtained in CVOTs in T2DM. However, these studies were designated application safety trials and not designed for CV or renal outcomes.

While many pathological mechanisms have similar initiation factors, like hyperglycaemia, others result in substantial differences between the two diseases, e.g. IR or obesity. For example, there is a strong association of the presence of IR and CKD.^126^ Resistance to the metabolic functions of insulin and compensatory hyperinsulinaemia has been shown to negatively impact kidney structure and function, via the RAS, the SNS, and other intrarenal mechanisms. This includes reductions in bioavailable NO and NO‐dependent vascular control, increased tissue inflammation, fibrosis, altered renal glomerular haemodynamics and impairment of tubuloglomerular feedback, hyperfiltration, and sodium retention.^126^ Furthermore, the development of DKD has been shown to be negatively impacted by the metabolic syndrome and individual components of the latter.^126^ All of these factors may contribute to early DKD in T2DM and be counteracted and partially prevented by the usage of SGLT‐2is, as discussed above. Early DKD in T1DM is initiated by hyperglycaemia and subsequent development of hyperfiltration by malfunction of the tubuloglomerular feedback, renal inflammation, and overactivation of renal RAS, as elucidated above. In both, T1DM and T2DM, increases in plasma uric acid were linked to negative renal outcomes. Also, obesity, the metabolic syndrome, or IR increase in prevalence in T1DM. This may be another reason to consider SGLT‐2is treatment in T1DM, as it not only impacts the individual components of the metabolic syndrome, but also the risk for DKA has been shown to be decreased in individuals with elevated BMI (BMI > 27 kg/m^2^).^127^

Current data on SGLT‐2i–mediated protective mechanisms range from systemic effects, like increased glycosuria and natriuresis resulting in decreased glucotoxicity, body weight, plasma volume, BP, and arterial stiffness, to direct cardiac and renal protective mechanisms like reduction in inflammation, oxidative stress, and many more, ultimately hypothesized to improve cardiac, vascular, and renal structure by decreasing incidence of cardiac and renal hypertrophy, thickening, stiffness and fibrosis.^18^, ^118^ Looking at available and emerging data, it is easy to point out obvious differences between onset and progression of DKD and CVD in T1DM and T2DM—when looking at the effects SGLT‐2is, it is unlikely that all mechanisms are identical as accounted for by different pathological mechanisms; however, net outcomes likely are going in a similar direction in primary and secondary prevention. SGLT‐2is have been shown to decrease hyperglycaemia to a similar extent in both T1DM and T2DM, improve insulin sensitivity in T2DM,^111^ promote weight loss and decrease tissue inflammation and subsequent effects on the kidneys. Furthermore, they were shown to modify renal (glomerular) haemodynamics and tubuloglomerular feedback in both T1DM and T2DM, albeit through different suggested mechanisms.^55^ Net effects seem to be a reduction in hyperglycaemia and associated effects, a reduction in glomerular hyperfiltration, and improvement of other downstream or associated pathological mechanisms, resulting in renal and cardiovascular protection. This was, for example, also shown by the DAPA‐HF trial which demonstrated that beneficial CV effects are independent of diabetes mellitus and hyperglycaemia.^128^

It remains to be elucidated, however, if described effects of SGLT‐2is are of comparable magnitude in the primary and/or secondary prevention of the comorbidities of the two different diseases. It may well be possible that due to longer exposure times or metabolic effects, SGLT‐2is may exert a stronger or weaker effect in T1DM. Either way, whether this is desirable has yet to be confirmed by observational studies in the long run.

## CONFLICT OF INTEREST

OS has acted as a member of advisory boards and given lectures for companies; and is CEO and founder of Sciarc GmbH. PV discloses the potential following conflict of interest: participation in expert committees for Astra Zeneca, Boehringer Ingelheim, Novo Nordisk, Daiichi‐Sankyo and Sanofi; lectures for Abbott, AstraZeneca, Bayer, Eli Lilly, Hikma, Merck‐Sharp‐Dohme, Novo Nordisk, Novartis, Pfizer and Sanofi; and research grants from Abbott, Bristol‐Myers‐Squibb‐AstraZeneca and Novo Nordisk. ES reports personal fees from Oxford Diabetes Trials Unit, AstraZeneca, Bayer, Berlin Chemie, Boehringer Ingelheim, Menarini, Merck Serono, Excemed, Novartis, Novo Nordisk and Sanofi. AC discloses the potential following conflict of interest: advisory board membership: Abbott, Astra Zeneca, Boehringer Ingelheim, DOC Generici, Eli Lilly, Janssen, Mundipharma, Novo Nordisk and OM Pharma; lectures for Astra Zeneca, Berlin Chemie, Boehringer Ingelheim, Eli Lilly, Mundipharma, Novo Nordisk and Roche Diagnostics; and research grants from Astra Zeneca, Eli Lilly, Mitsubishi and Novartis.

## AUTHORS’ CONTRIBUTIONS

OS, ES, PV and AC supported the content and writing of the white paper. All authors read and approved the final manuscript.

## Data Availability

Data sharing not applicable to this article as no data sets were generated during the current study.
